# Notes on the *Nazeris* fauna of Yunnan Province, China (Coleoptera, Staphylinidae, Paederinae)

**DOI:** 10.3897/zookeys.84.1189

**Published:** 2011-03-01

**Authors:** Jia-Yao Hu, Li-Zhen Li, Yun-long Zhao

**Affiliations:** 1 Department of Biology, East China Normal University, 3663 North Zhongshan Road, Shanghai, 200062 P. R. China; 2 Department of Biology, Shanghai Normal University, 100 Guilin Road, Shanghai, 200234 P. R. China

**Keywords:** Coleoptera, Staphylinidae, Paederinae, *Nazeris*, key, Yunnan, China, new species

## Abstract

Two new species of the genus Nazeris Fauvel collected from Nabanhe Nature Reserve, Yunnan Province, are described under the names of Nazeris nabanhensis **sp. n.** and Nazeris caoi **sp. n.** The male sexual characters are described and illustrated. A key to the Nazeris species of Yunnan is provided. A map of the collecting sites is given.

## Introduction

The genus Nazeris Fauvel (1873: 298) can be readily distinguished from other Paederinae by the labrum having four teeth at the front margin and the bi-lobed 4th tarsal segments. Up to the present, 51 species and subspecies of Nazeris have been recorded from China. Yunnan is a mountainous province located in Southwest China, from which nine species of Nazeris have been described: Nazeris zhangi Watanabe & Xiao (1993: 130) from “Yu’an-shan near Kunming City”, Nazeris giganteus Watanabe & Xiao (1997:2) and Nazeris daliensis Watanabe & Xiao (1997: 7) from “Diancang shan Mts., Dali shi”, Nazeris alpinus Watanabe & Xiao (1997: 5) from “Mt. Yulongxue shan, Lijiang County”, Nazeris jizushanensis Watanabe & Xiao (1997: 9) from “Mt. Jizu Shan, Binchuan County”, Nazeris baihuaensis Watanabe & Xiao (2000: 312), Nazeris ishiianus Watanabe & Xiao (2000: 319) and Nazeris nomurai Watanabe & Xiao (2000: 316) from “Gaoligong Shan Mts., Baoshan area”, Nazeris huanxipoensis Watanabe & Xiao (2000: 318) from “Huanxipo, Tengchong Xian”. Only two species have become known from the three countries adjacent to Yunnan (Myanmar, Laos and Vietnam): Nazeris coomani Jarrige (1948: 40) (redescribed by Rougemont 1988: 775) from “du mont Bavi, Tonkin” (Vietnam) and Nazeris odzisan Watanabe (1996: 1) from “Mt. Tam Dao, Vinh Phu Prov.” (Vietnam).

The specimens from Yunnan Province contained another two undescribed species, Nazeris nabanhensis sp. n. and Nazeris caoi sp. n. The male sexual characters of the two new species are described and illustrated. A map (fig. 13) of the collecting sites of Nazeris species in Yunnan and a key to the Yunnan species are provided.

The types are deposited in the Insect Collections of Department of Biology, Shanghai Normal University, Shanghai, P. R. China (SHNUC).

## Methods

The specimens were collected from decaying leaf litter of forest floors by hand sifting. They were killed with ethyl acetate and dried. To examine the male genitalia, the last four abdominal segments were detached from the body after softening in hot water. The aedeagi and sternites were mounted in Euparal on plastic slides. Drawings were made using an Olympus SZ61 microscope. Photos were taken with an Olympus E420 camera mounted on an Olympus SZX12 stereoscope. Material of other Yunnanese Nazeris species was not examined. The characters used in comparative remarks and keys are according to descriptions of (Watanabe and Xiao (1993, 1997, 2000), Watanabe (1996), Jarrige (1948) and Rougemont (1988).

### Measurements

Body length: measured from anterior margin of labrum to end of abdomen;

Forebody length: measured from anterior margin of labrum to elytral apices;

Eye length: longitudinal length of eye in dorsal view;

Postocular length: length of postocular portion in dorsal view;

Head width: width of head across eyes;

Pronotum width: width of pronotum across the widest part;

Elytra width: width of elytra across the widest part;

Head length: measured from front margin of head to its posterior margin;

Pronotum length: measured from front margin of pronotum to its posterior margin;

Elytra length: measured from humeral angle to apicolateral angle.

## Descriptions

### Nazeris nabanhensis sp. n.

urn:lsid:zoobank.org:act:7B0F8A68-2EEE-412E-9090-9F1CE8EBA8ED

Figs. 1 3–7 

#### Type material.

CHINA: Holotype: Yunnan Prov.: male, Jinghong City, Nabanhe Nature Reserve, Benggangxinzhai, 1,750m, 16. XI. 2008, Hu Jia-Yao & Tang Liang leg. Paratypes: 2 females, Jinghong City, Nabanhe Nature Reserve, Bengganghani, Nanmugahe, 1,700m, 11. XI. 2008, Hu Jia-Yao & Tang Liang leg.; 1 female, Jinghong City, Nabanhe Nature Reserve, Bengganghani, 1,800m, 14. XI. 2008, Hu Jia-Yao & Tang Liang leg.; 5 males, 8 females, same locality as holotype, 3. V. 2009, Hu Jia-Yao & Yin Zi-Wei leg.; 1 female, Jinghong City, Nabanhe Nature Reserve, Bengganghani, Nanmugahe, 1,700m, 27. IV. 2009, Hu Jia-Yao & Yin Zi-Wei leg.; 3 females, Jinghong City, Nabanhe Nature Reserve, Bengganghani, Chuguohe, 1,700m, 28. IV. 2009, Hu Jia-Yao & Yin Zi-Wei leg.; 1 male, 1 female, Jinghong City, Nabanhe Nature Reserve, Bengganghani, 1,650m, 29. IV. 2009, Hu Jia-Yao & Yin Zi-Wei leg.; 1 male, Jinghong City, Nabanhe Nature Reserve, Bengganghani, Nanmugahe, 1700m, 30. IV. 2009, Hu Jia-Yao & Yin Zi-Wei leg.; 4 females, Jinghong City, Nabanhe Nature Reserve, Bengganghani, 1,650m, 30. IV. 2009, Hu Jia-Yao & Yin Zi-Wei leg. SHNUC.

**Figures 1–2.**
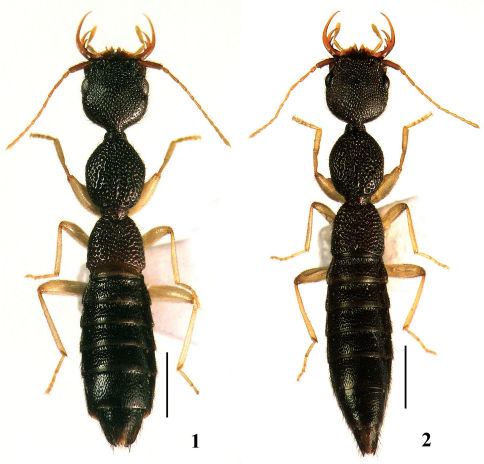
Habitus of Nazeris. **1** Nazeris nabanhensis sp. n. **2** Nazeris caoi sp. n. Scale bars 1 mm.

#### Description.

Body length: 5.8–6.4 mm; forebody length: 3.3–3.5 mm.

##### Male.

###### Body

(Fig. 1) elongate, dark brown, with labrum, coxae, and basal antennomeres reddish yellow, the remaining antennomeres, maxillary palpi and legs yellow, with exception for coxae.

###### Head

suborbicular, longer than wide (length/width = 1.18); postocular portion 1.96 times as long as eye length; punctation coarse, dense, and umbilicate; interstices reduced to narrow ridges. Antennae slender; relative length of each segment from 1 to 11: 42.0 : 15.0 : 29.0 : 23.0 : 22.5 : 22.0 : 19.0 : 17.0 : 15.5 : 14.0 : 20.0; relative width of each segment from 1 to 11: 12.0 : 7.5 : 6.0 : 6.0 : 6.0 : 6.0 : 6.0 : 6.0 : 6.0 :7.5 : 7.5.

###### Pronotum

convex, oval, longer than wide (length/width = 1.20), narrower (pronotum/head = 0.93) and shorter (pronotum/head = 0.94) than head; prosternum with strong longitudinal median carina, which disappears behind anterior margin. Elytra shorter than wide (length/width = 0.91), distinctly shorter (elytra/pronotum = 0.74) and slightly narrower (elytra/pronotum = 0.97) than pronotum.

###### Abdomen

**Figures 3–7.**
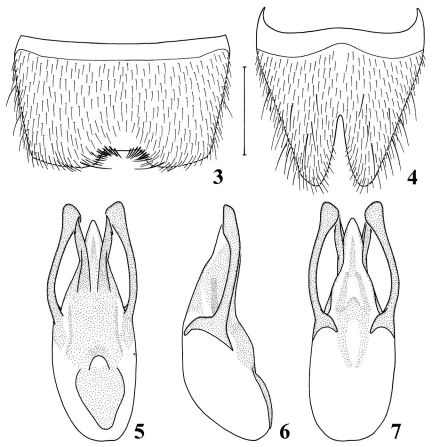
Details of Nazeris nabanhensis sp. n. **3** male 7th sternite **4** male 8th sternite **5** aedeagus, in dorsal view **6** aedeagus, in lateral view **7** aedeagus, in ventral view. Scale bar: 0.5 mm.

elongate, tergites without any microsculpture. Seventh sternite (Fig. 3) trapezoidally emarginated in middle of posterior margin and distinctly depressed in front of emargination; 8th sternite (Fig. 4) V-shaped deeply excised in middle of posterior margin. Aedeagus (Figs. 5, 6 and 7) well sclerotized; apical part of median lobe in dorsal view tri-lobed, median part cone-shaped, two outer parts acute at apices, with little agnail in each outer side near apex; dorso-lateral apophyses slightly curved inward, distinctly widened near apex, extending beyond apices of median lobe.

##### Female.

Seventh and 8th sternites simple. The other characters are similar to those of male.

#### Remarks.

The new species is similar to Nazeris daliensis Watanabe (1997: 7) from Yunnan Province in appearance, but it can be distinguished from the latter by the following characters: elytra slightly narrower than pronotum (in Nazeris daliensis nearly as wide as, or slightly broader than pronotum); depth of excision of male 8th sternite nearly half of middle length of sternite (in Nazeris daliensis much shallower, nearly 1/3 of middle length of 8th sternite); apical part of median lobe of aedeagus in dorsal view tri-lobed (in Nazeris daliensis not lobed). The new species can be distinguished from Nazeris coomani Jarrige (1948: 40) from Vietnam by head with umbilicate punctation (in Nazeris coomani punctation of head simple) and distinguished from Nazeris odzisan Watanabe (1996: 1) from Vietnam by elytra shorter than wide (in Nazeris odzisan elytra longer than wide); dorso-lateral apophyses of aedeagus extending beyond apices of median lobe (in Nazeris odzisan not extending to apices of median lobe).

#### Etymology.

The specific name is derived from the name of the type locality: Nabanhe Nature Reserve.

### Nazeris caoi sp. n.

urn:lsid:zoobank.org:act:3B251645-1B6B-4388-ACEC-4E5B42C4A858

Figs. 2 8–12 

#### Type material.

CHINA: Holotype: Yunnan Prov.: male, Jinghong City, Nabanhe Nature Reserve, Bengganghani, 1,930m, 14. XI. 2008, Hu Jia-Yao & Tang Liang leg. Paratypes 1 male, same data as holotype; 1 female, Jinghong City, Nabanhe Nature Reserve, Bengganghani, 1,900m, 1. V. 2009, Hu Jia-Yao & Yin Zi-Wei leg. SHNUC.

#### Description.

Body length: 6.1–6.4 mm; forebody length: 3.3–3.6 mm.

##### Male.

###### Body

(Fig. 2) elongate, dark brown, with labrum, coxae, and basal two antennomeres reddish yellow, the remaining antennomeres, maxillary palpi and legs yellow, with exception for coxae.

###### Head

suborbicular, slightly longer than wide (length/width = 1.07); postocular portion 2.14 times as long as eye length; punctation coarse, dense, and umbilicate; interstices reduced to narrow ridges. Antennae slender; relative length of each segment from 1 to 11: 42.0 : 13.5 : 30.0 : 23.0 : 21.0 : 21.0 : 19.0 : 18.0 : 18.0 : 16.0 : 22.0; relative width of each segment from 1 to 11: 10.5 : 7.0 : 6.5 : 6.0 : 5.5 : 6.0 : 5.5 : 5.5 : 6.0 :6.5 : 7.0.

###### Pronotum

convex, oval, longer than wide (length/width = 1.19), narrower (pronotum/head = 0.88) and shorter (pronotum/head = 0.98) than head; prosternum with strong longitudinal median carina, which disappears behind anterior margin. Elytra slightly shorter than wide (length/width = 0.97), distinctly shorter (elytra/pronotum = 0.80) and slightly narrower (elytra/pronotum = 0.97) than pronotum.

###### Abdomen

**Figures 8–12.**
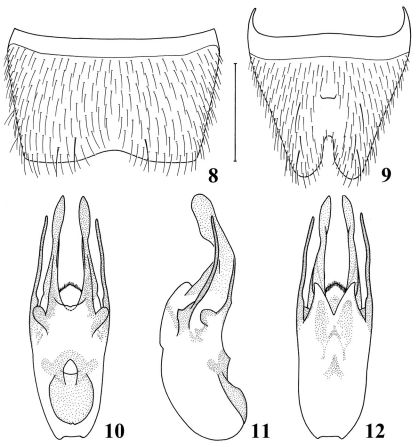
Details of Nazeris caoi sp. n. **8** male 7th sternite **9** male 8th sternite **10** aedeagus, in dorsal view **11** aedeagus, in lateral view **12** aedeagus, in ventral view. Scale bar: 0.5 mm.

elongate, tergites without any microsculpture; densely and coarsely punctate. Seventh sternite (Fig. 8) distinctly emarginated in middle of posterior margin; 8th sternite (Fig. 9) with little short protrusion in middle, deeply excised in middle of posterior margin. Aedeagus (Figs. 10, 11 and 12) well sclerotized; median lobe bi-lobed in dorsal view, curved ventrad in apical 1/3 in lateral view; dorso-lateral apophyses very thin, slightly curved ventrad, not extending to apices of median lobe.

##### Female.

Seventh and 8th sternites simple. The other characters are similar to those of male.

#### Remarks.

The present new species is similar in appearance to Nazeris nabanhensis sp. n. from the same locality, but can be distinguished from the latter by the following characters: postocular part more than twice as long as longitudinal diameter of each eye (in Nazeris nabanhensis less than twice as long as longitudinal diameter of each eye); median lobe of aedeagus in dorsal view bi-lobed (in Nazeris nabanhensis tri-lobed); dorso-lateral apophyses of aedeagus not extending to apices of median lobe (in Nazeris nabanhensis extending beyond apices of median lobe). The new species can be distinguished from Nazeris coomani Jarrige (1948: 40) from Vietnam by head with umbilicate punctation (in Nazeris coomani punctation of head simple), and distinguished from Nazeris odzisan Watanabe (1996: 1) from Vietnam by elytra shorter than wide (in Nazeris odzisan elytra longer than wide); median lobe of aedeagus in dorsal view bi-lobed (in Nazeris odzisan not lobed).

#### Etymology.

The species is named in honor of Mr. Guanghong Cao of Nabanhe Nature Reserve, who helped us a lot during field work.

**Figures 13.**
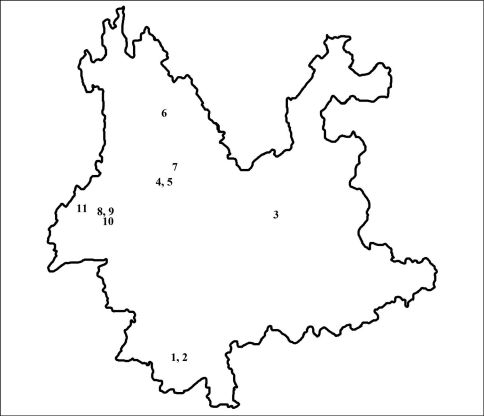
Map showing the collecting sites of the Nazeris in Yunnan Prov.; **1** Nazeris nabanhensis sp. n. **2** Nazeris caoi sp. n. **3** Nazeris zhangi Watanabe & Xiao **4** Nazeris giganteus Watanabe & Xiao **5** Nazeris daliensis Watanabe & Xiao **6** Nazeris alpinus Watanabe & Xiao **7** Nazeris jizushanensis Watanabe & Xiao **8** Nazeris baihuaensis Watanabe & Xiao **9** Nazeris ishiianus Watanabe & Xiao **10** Nazeris nomurai Watanabe & Xiao **11** Nazeris huanxipoensis Watanabe & Xiao.

### Key to species of Nazeris from Yunnan Province, China

**Table:** 

1	Body length less than 4.5 mm; posterior margin of male 7th sternite not emarginate	Nazeris zhangi Watanabe & Xiao
–	Body length at least 5.0 mm; posterior margin of male 7th sternite emarginate	2
2	Male 8th sternite with short protrusion in middle, median lobe of aedeagus bi-lobed in dorsal view	Nazeris caoi sp. n.
–	Male 8th sternite without protrusion in middle, median lobe of aedeagus not bi-lobed in dorsal view	3
3	Median lobe of aedeagus tri-lobed in dorsal view	Nazeris nabanhensis sp. n.
–	Median lobe of aedeagus not tri-lobed in dorsal view	4
4	Dorso-lateral apophyses of aedeagus extending beyond apex of median lobe	5
–	Dorso-lateral apophyses of aedeagus not extending to apex of median lobe	9
5	Dorso-lateral apophyses of aedeagus dilated in apical part and markedly curved ventrad in apical half	Nazeris nomurai Watanabe & Xiao
–	Dorso-lateral apophyses of aedeagus not dilated in apical part and not markedly curved ventrad in apical half	6
6	Median lobe of aedeagus subtriangular in apical third and curved dorsad near apex in lateral view, dorso-lateral apophyses curved ventrad near apex in lateral view	Nazeris huanxipoensis Watanabe & Xiao
–	Median lobe of aedeagus not subtriangular in posterior third, not curved dorsad near apex in lateral view, dorso-lateral apophyses nearly straight in lateral view	7
7	Dorso-lateral apophyses of aedeagus dilated at middle; median lobe of aedeagus without wing-shaped process	Nazeris baihuaensis Watanabe & Xiao
–	Dorso-lateral apophyses of aedeagus not dilated at middle; median lobe of aedeagus with a wing-shaped process on each side near middle	8
8	Postocular part less than twice as long as eye length; depth of excision of posterior margin of male 8th sternite more than twice its width	Nazeris jizushanensis Watanabe & Xiao
–	Postocular part more than twice as long as eye length; depth of excision of posterior margin of male 8th sternite nearly the same its width	Nazeris daliensis Watanabe & Xiao
9	Median lobe of aedeagus semicircularly emarginate at apex	Nazeris ishiianus Watanabe & Xiao
–	Median lobe of aedeagus not emarginate at apex	10
10	Median lobe of aedeagus tongue-shaped; dorso-lateral apophyses of aedeagus very narrow and near straight in dorsal view	Nazeris alpinus Watanabe & Xiao
–	Median lobe of aedeagus not tongue-shaped, distinctly narrowed in apical 1/4 in dorsal view; dorso-lateral apophyses of aedeagus markedly curved inward in dorsal view	Nazeris giganteus Watanabe & Xiao

## Supplementary Material

XML Treatment for Nazeris nabanhensis

XML Treatment for Nazeris caoi
